# Experimental demonstration of anomalous Floquet topological insulator for sound

**DOI:** 10.1038/ncomms13368

**Published:** 2016-11-11

**Authors:** Yu-Gui Peng, Cheng-Zhi Qin, De-Gang Zhao, Ya-Xi Shen, Xiang-Yuan Xu, Ming Bao, Han Jia, Xue-Feng Zhu

**Affiliations:** 1 School of Physics, Huazhong University of Science and Technology, Luoyu road No. 1037, Wuhan, Hubei 430074, China; 2 Innovation Institute, Huazhong University of Science and Technology, Wuhan 430074, China; 3 Key Laboratory of Noise and Vibration Research, Institute of Acoustics, Chinese Academy of Sciences, Beijing 100190, China; 4 School of Automation, Wuhan University of Technology, Wuhan 430063, China

## Abstract

Time-reversal invariant topological insulator is widely recognized as one of the fundamental discoveries in condensed matter physics, for which the most fascinating hallmark is perhaps a spin-based topological protection, the absence of scattering of conduction electrons with certain spins on matter surface. Recently, it has created a paradigm shift for topological insulators, from electronics to photonics, phononics and mechanics as well, bringing about not only involved new physics but also potential applications in robust wave transport. Despite the growing interests in topologically protected acoustic wave transport, *T*-invariant acoustic topological insulator has not yet been achieved. Here we report experimental demonstration of anomalous Floquet topological insulator for sound: a strongly coupled metamaterial ring lattice that supports one-way propagation of pseudo-spin-dependent edge states under *T*-symmetry. We also demonstrate the formation of pseudo-spin-dependent interface states due to lattice dislocations and investigate the properties of pass band and band gap states.

## Abstract (web summary)

Topological protected acoustic wave propagation has been predicted, but yet awaits for experimental demonstration. Here, Peng *et al*. report one-way propagation of pseudo-spin-dependent edge states for sound, analogous to Floquet topological insulator in solid state.

## Graphical abstract

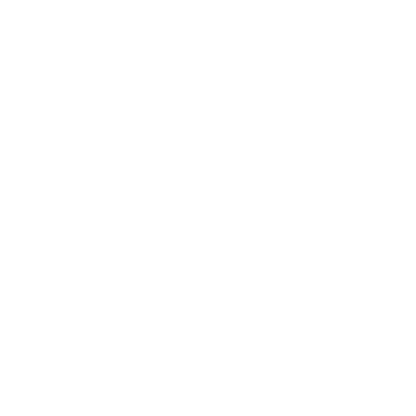

The study of topological properties or invariants in periodic physical systems has become one of the most active fields in many branches of physics^1^,^2^,^3^,^4^,^5^,^6^,^7^,^8^,^9^,^10^,^11^,^12^,^13^,^14^,^15^,^16^,^17^,^18^,^19^,^20^. As basic elements of topological band theory, these topological invariants are very interesting, because they imply the presence of non-trivial bulk topologies, giving rise to the presence of gapless edge or surface states featured with the absence of scatterings. Probably the most well-known example should be the integer quantum Hall effect discovered by Klitzing *et al*.^21^ under strong magnetic fields and low temperatures, where the quantized Hall conductivity is insensitive to local perturbations and the quantization (TKNN integer) was afterwards discovered to have an expression of the first Chern number of the bands below the chemical potential^22^. Another seminal work is the Kane and Mele model, dealing with time-reversal (*T*) invariant systems of strong spin–orbit couplings^23^. In that work, Kane and Mele^23^ introduce a distinctive *Z*_2_ index to describe the *T*-invariant quantum spin Hall phase that has a spin-dependent topological protection and robustness against non-magnetic disorder and interactions under *T*-symmetry. The new topological phase in the presence of *T*-symmetry was later termed topological insulators, now widely recognized as a fundamental discovery in condensed matter physics^1^,^2^,^3^,^4^,^5^.

In the past decade, exploration for new types of topological insulators is substantively followed up in different subfields of physics, making a paradigm shift for topological states, from electronics to photonics^6^,^7^,^8^,^9^,^10^,^11^,^12^,^24^,^25^, phononics^13^,^14^ as well as mechanics^15^,^16^,^17^. In photonics, the field of topological insulators is rapidly developing in the past few years, where one of most representative example should be the Floquet topological insulator, proposed in periodically driven systems. There are basically two types of Floquet topological insulator. One is based on temporal modulation^26^. As the time reversal symmetry is broken, such topological insulator can support non-trivial one-way edge states that are immune to disorder-induced backscattering. The other relies on the three-dimensional chiral couplings^9^, where the *z* direction wave evolution can be mapped into the temporal modulation in the *x*–*y* plane. In reality, it is quite challenging to realize very fast temporal modulation or fabricate delicate three-dimensional helical waveguide array on chip. A more attractive solution is proposed by Hafezi *et al*.^7^,^8^ employing a two-dimensional (2D) coupled resonator optical waveguide (CROW) lattice to exhibit an optical analoge of quantum spin Hall effect. In stark contrast with aforementioned two types of Floquet topological insulator, the CROW lattice is a 2D time-reversal invariant system, which does not require any type of external driven and can be easily demonstrated in experiments. The CROW lattice, for which the configuration is specifically designed to be periodic, is termed as anomalous Floquet topological insulator (AFI)^11^,^12^ and has zero Chern number due to the time-reversal symmetry. In acoustics, unidirectional edge channels have been recently proposed for scalar acoustic waves propagating in a fluid circulation array^18^,^27^,^28^, mimicking the integer quantum Hall effect by breaking *T*-symmetry via the Doppler effect. However, the missing part corresponding to *T*-invariant topological phase for sound has still not yet been achieved.

In this study, we have theoretically proposed and experimentally demonstrated the prototype of *T*-invariant AFI for sound. The proposed AFI is inspired from the Chalker–Coddington network model developed in the 1980s for the study of Anderson transition in quantum Hall systems^29^. The model of AFI is a 2D coupled metamaterial ring lattice, presented in Fig. 1a, which can support topological edge states at sufficiently large coupling strength between neighbouring lattice rings. Figure 1b schematically shows a unit-cell of the 2D lattice, comprising one lattice ring in the centre surrounded by four coupling rings. Here we define a pseudo-spin for acoustic waves based on wave circulation direction in the lattice rings^7^,^8^,^11^,^30^, *viz.* pseudo-spin-up↔clockwise and pseudo-spin-down↔anti-clockwise. By purposely designing the lattice configuration, we have successfully constructed the acoustic AFI, which can support the pseudo-spin-dependent edge states, scattering immune to boundary abrupt variations or lattice dislocations. The edge states with different pseudo-spins can propagate in opposite directions under *T*-symmetry at the same boundary. We emphasize that the edge states with different pseudo-spins are regarded as decoupled due to nearly unitary coupling between neighbouring rings, closing the time-reversed channels for backscatterings. In this case, the rings no longer act as resonators and the edge state is essentially a conventional waveguide mode^31^ (see Supplementary Note 1). For one specific pseudo-spin, there exists a pair of edge states propagating along the upper and lower boundaries, respectively, with opposite group velocities^7^,^8^,^11^,^24^. The observations reveal that when the coupling strength between adjacent lattice rings surpasses a threshold, acoustic waves carrying a pseudo-spin in one lattice ring may tunnel into the neighbouring coupling ring with the pseudo-spin flipped and then go to another lattice ring with the pseudo-spin restored or conserved, rendering an interesting zigzag route. Our work represents an important step in the implementation of a diverse family of topological structures and networks for sound with new properties and functionalities.

## Results

### Projected band structure for acoustic AFI

**Figure 1:**
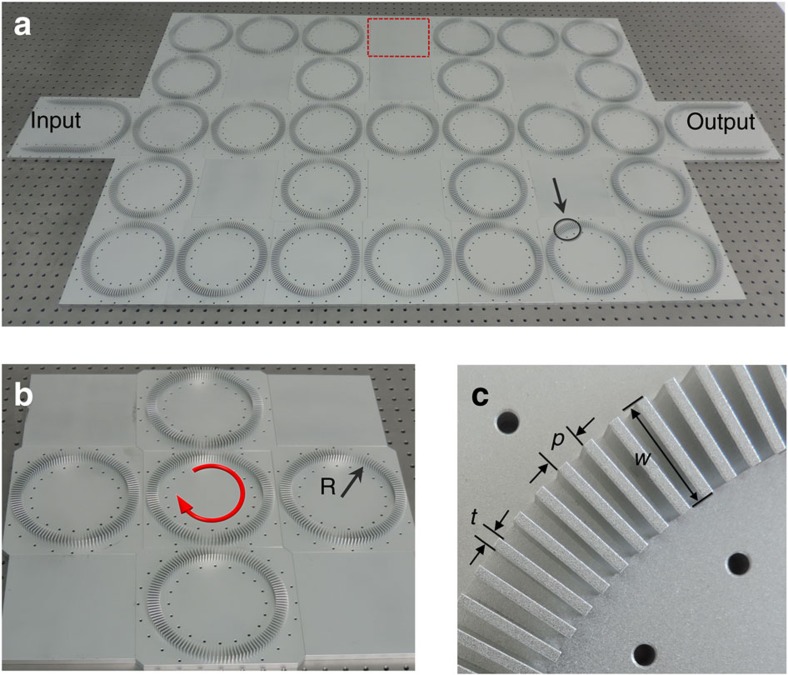
Photograph of the fabricated samples. (**a**) Photograph of a 2D coupled metamaterial ring lattice composed of 3 × 4 unit cells. The U-shape waveguides are the input and output ports for selectively exciting and extracting edge states with specific pseudo-spins. It is noteworthy that a ring is purposely removed to form a defect, as marked by the red dashed box. (**b**) Photograph of a unit cell composed of one lattice ring surrounded by four coupling rings with the radius *R*=77.4 mm. The red arrow shows that the waves in the lattice ring are carrying pseudo-spin-up, *viz*. propagating in clockwise. (**c**) Zoom-in photograph of the metamaterial waveguide marked by the black circle in **a**. The period of the waveguide is *P*=4.3 mm. The thickness and width of the metal plate is *t*=1.7 mm and *w*=17.2 mm.

As shown in Fig. 1c, our acoustic metamaterial waveguide is constructed by subwavelength air–metal layers (metal: aluminum alloys) periodically stacking along the azimuthal direction of the ring. The effective refractive index of the fundamental guided mode can be expressed as^32^,^33^


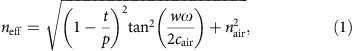


where *t*, *w* are the thickness and width of metal plates, *p* is the structural period. *ω*=2*πf* denotes the angular frequency of acoustic waves. Here, the refractive index of air *n*_air_=1 and the speed of sound in air at room temperature *c*_air_=343.21 m s^−1^. For the fabricated sample, the structural parameters are *R*=77.4 mm, *w*=17.2 mm, *t*=1.7 mm and *P*=4.3 mm. In stark contrast to the natural existing materials with refractive indices always lower than *n*_air_^34^, the extremely anisotropic metamaterial can support tightly guided acoustic waves, as the effective refractive index is much larger than *n*_air_. To suppress high-order guided modes, the operation frequency should satisfy the cutoff condition of *ω*<*πc*_air_/*w.*

**Figure 2:**
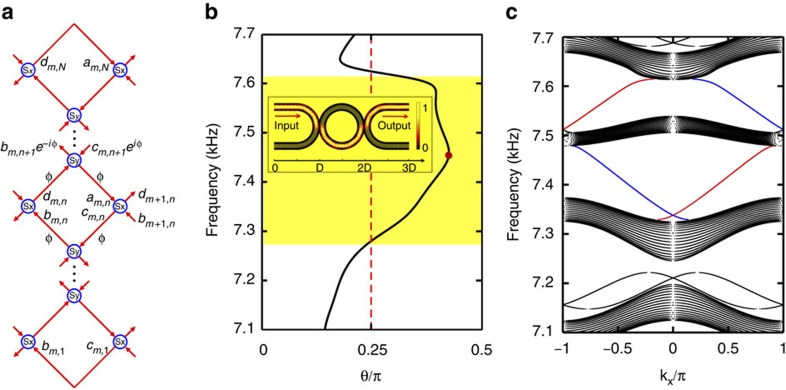
Projected band structure for acoustic AFI. (**a**) The equivalent Chalker–Coddington network model of the coupled ring lattice that is periodic in the *x* direction and finite in the *y* direction with *N* unit cells. Red arrows refer to the links, *viz*. quarter lattice rings, and blue circles refer to the nodes, *viz*. coupling rings. (**b**) The coupling strength *θ* between neighbouring lattice rings as the frequency varies from 7.1 to 7.7 kHz. The yellow region covers the frequency range of strong coupling for *θ*>*π*/4. The inset shows the simulated sound pressure amplitude distribution in the U-Ring-U shape waveguide as *θ* reaches maximum *θ*≈0.43*π* at 7.46 kHz. (**c**) Projected band structure of the semi-infinite ring lattice (*N*=20) for pseudo-spin-up Bloch modes. The red and blue bands denote non-trivial edge states at the upper and lower boundaries of the lattice.

For calculating the projected band structure of AFI, we first map the lattice onto the equivalent Chalker–Coddington network model composed of links and nodes^28^,^29^. As shown schematically in Fig. 2a, each red arrow refers to a link, *viz*. a quarter lattice ring, and each blue circle refers to a node, *viz*. a coupling ring. In the link, the phase delay of acoustic waves is *ϕ*=(*πR*/2)*ωn*_eff_/*c*_air_, which is dependent on the operation frequency. Each node can be described by a scattering matrix in a parameterization form refs 11, 30


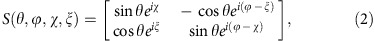


where *θ*∈[0, *π*/2] characterizes the coupling strength between adjacent lattice rings. *χ*, *ϕ* and *ξ*∈[0, 2*π*] are the phase parameters to be determined. As the coupling ring is symmetric under 180° rotation, the scattering matrix should satisfy *S*_11_=*S*_22_ and *S*_12_=*S*_21_, that is, *e*^*iχ*^=*e*^*i*(*ϕ*−*χ*)^ and *e*^*iξ*^=−*e*^*i*(*ϕ*−*ξ*)^. Therefore, we obtain *χ*=*ϕ*/2 and *ξ*=(*π*+*ϕ*)/2. The scattering matrix can thus be rewritten into


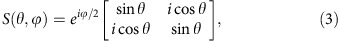


where *ϕ* denotes the phase shift in the coupling process. As *ϕ* has no influence on the coupling strength, it can be arbitrarily chosen. For simplicity, we set *ϕ*=0 and obtain


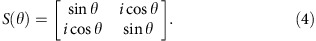


From equation (4), the scattering matrix *S* is only determined by *θ*, which characterizes the coupling strength between adjacent lattice rings. It should be mentioned that the coupling strength *θ* is highly dispersive in our case, whereas in previous theoretical works^11^,^30^, *θ* has always been considered as non-dispersive for the calculation of 'quasi-energy' band structures (see Supplementary Fig. 1). In Fig. 2a, we consider a strip coupled ring lattice that is periodic in *x* direction and finite in *y* direction. The sites of lattice rings are indicated by (*m*, *n*), where *m* and *n* are the column and row indices, respectively. Then, we employ |*ϕ*_*m*,*n*_〉=[*a*_*m*,*n*_ *b*_*m*,*n*_ *c*_*m*,*n*_ *d*_*m*,*n*_]^*T*^ to represent the amplitudes of acoustic waves in the lattice ring at the site (*m*, *n*). The scattering matrixes of coupling rings in *x* and *y* directions are the same *S*_*x*_=*S*_*y*_=*S*(*θ*). Coupling relations between the lattice rings at sites (*m*, *n*) and (*m*+1, *n*) in *x* direction, as well as those at sites (*m*, *n*) and (*m*, *n*+1) in *y* direction are respectively expressed as follows


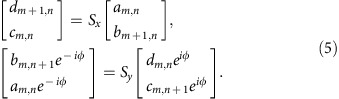


By applying the Bloch condition in *x* direction |*ϕ*_*m*+1,*n*_〉=*e*^*iKx*^|*ϕ*_*m*,*n*_〉 and the boundary condition in *y* direction *c*_*m*,1_=*e*^−2i*ϕ*^*b*_*m*,1_, *a*_*m*,*N*_=*e*^2*iϕ*^*d*_*m*,*N*_, we can obtain the following governing equation^11^


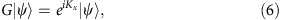


where *K*_*x*_ is the Bloch wave vector, |*ψ*〉=[*b*_*m*,1_ *d*_*m*,1_ *b*_*m*,2_ *d*_*m*,2_ ··· *b*_*m*,*N*_ *d*_*m*,*N*_]^*T*^ the wave amplitudes in the *m*th column, *G*=*G*_1_·*G*_2_ with *G*_1_ and *G*_2_ being


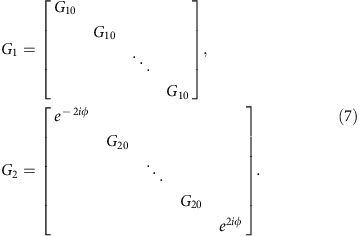


The sub-matrixes *G*_10_ and *G*_20_ are


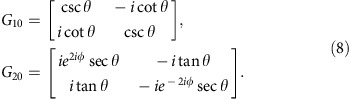


Figure 2b has clearly illustrated the coupling strength *θ* between neighbouring lattice rings as the frequency *f* varies from 7.1 to 7.7 kHz. The parameter *θ*=arcsin(|*P*_out_|/|*P*_in_|) is numerically extracted from the U-Ring-U shape configuration by using a finite element solver (COMSOL Multiphysics TM 4.3b), where *P*_in_ and *P*_out_ are the pressure amplitudes at input and output ports, respectively. The yellow region in Fig. 2b covers the frequency range of strong coupling for *θ*>*π*/4 and the coupling strength reaches a maximum *θ*≈0.43*π* at 7.46 kHz. The inset has shown the simulated pressure amplitude distribution at 7.46 kHz to validate the nearly unitary coupling.

In Fig. 2c, we map out the projected band structure for pseudo-spin-up Bloch modes in the semi-infinite ring lattice by solving the governing equation (6). Specifically, the number of unit cells in *y* direction is set as *N*=20 and the band structure is plotted by sweeping the frequency from 7.1 to 7.7 kHz with the step of Δ*f*=0.1 Hz. Each dot in the bands corresponds to a Bloch mode with an explicit frequency *f* and a Bloch wave vector *k*. In Supplementary Fig. 2, we also show the band structures calculated by different frequency steps, *viz*., Δ*f*=1, 0.1 and 0.025 Hz, which clearly shows that the central blank parts of the band structure in Fig. 2c are shrinking by decreasing the frequency step. In the strong coupling range for *θ*>*π*/4, our system may turn into a topological insulator. It should be mentioned that *θ*>*π*/4 is a necessary but not sufficient condition to judge whether the coupled ring lattice is a topological insulator. Therefore, the yellow region in Fig. 2b just shows the frequency range where the ring lattice may possibly be a topological insulator. To accurately predict the frequency range of topological phase, we need to refer to the projected band structure plotted in Fig. 2c. We note that the topological insulator studied in refs 11, 12, 30 also has bulk state bands and gapless edge state bands at *θ*>*π*/4. In Fig. 2c, the red and blue curves denote the upper and lower edge states spanning the band gaps with positive and negative group velocities, respectively. Obviously, the non-trivial edge states exist in the frequency range of 7.4∼7.6 kHz, showing a good agreement with the shaded region in Fig. 2b. However, in the weak coupling range for *θ*<*π*/4, the lattice turns into a trivial insulator supporting two-way edge states. To be specific, at the frequency around 7.2 kHz, there are actually two pseudo-spin-up edge states with different group velocities for either boundary. In Fig. 2c, we have neglected the pseudo-spin-flipping processes at each coupling connection. If we consider both pseudo-spins, the projected band structure will have a time-reversal counterpart^11^. For a certain pseudo-spin, the Berry curvature *F*(***k***) is an odd function in momentum space (or ***k***-space). Therefore, the Chern number *C* is calculated to be zero according to the definition of 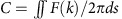, where the surface integral is operated for a specific bulk band in the Brillouin zone. According to refs 11, 30, we can use the none-zero *ν*_1_ invariant to describe the non-trivial topological properties of time-reversal invariant systems, for example, AFI, even though the Chern number is zero. The non-zero *ν*_1_ is defined by


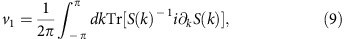


where the Bloch vector *k* is integrated over the first Brillouin zone, see Supplementary Fig. 3 and Supplementary Note 2.

### Coupling in the U-Ring-U shape waveguide complex

**Figure 3:**
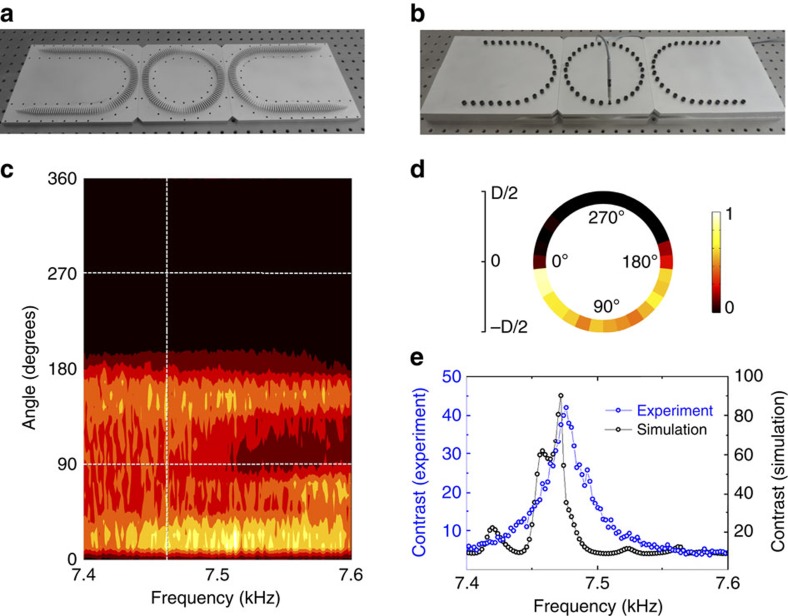
Coupling in the U-Ring-U shape waveguide complex. (**a**) Photograph of the U-Ring-U shape metamaterial waveguide complex. The structure is a four-port system with two coupling connections marked by the red elliptical circles. (**b**) Illustration of the experimental measurement setup. The metamaterial waveguide is sealed in rigid rectangular pipes to eliminate radiation losses. A condenser microphone is inserted into perforated holes on the pipe wall, to measure the pressure amplitude in the metamaterial waveguides. (**c**) Measured pressure amplitude distributions in the coupling ring from 7.4 to 7.6 kHz. (**d**) The measured pressure amplitude distribution in the coupling ring at 7.46 kHz, replotted from the data on the vertical dashed line in **c**. (**e**) The measured and simulated pressure amplitude contrast at 90° and 270° positions in the coupling ring from 7.4 to 7.6 kHz, where the measured contrast is replotted from the data on the horizontal dashed lines in **c**.

To demonstrate the coupling properties between neighbouring lattice rings in the strong coupling regime, we experimentally study a U-Ring-U shape waveguide complex displayed in Fig. 3a, which serves as the fundamental element of AFI. In the waveguide complex, the lattice rings are replaced by U-shape waveguides, thus forming a four-port system described by a scattering matrix *S*(*θ*). The coupling between two U-shape waveguides is realized via two connections, as marked by the red elliptic circles. The experimental setup is shown in Fig. 3b, where the fabricated U-Ring-U shape waveguide is sealed in rigid rectangular pipes to prevent unwanted radiation losses during propagation and isolate ambient noises. In the experiment, we launch acoustic waves from Port 1 and measure pressure amplitude distributions in the coupling ring by inserting a condenser microphone into holes perforated on the pipes. Meanwhile, we plug the unmeasured holes with screws.

Figure 3c has shown pressure amplitude distribution in the coupling ring along the azimuthal direction from 0° to 360°. The frequency range is taken from 7.4 to 7.6 kHz, which corresponds to the non-trivial band gap in Fig. 2c. It is observed that the sound energy is mainly distributed along the lower half ring, leaving the upper half part almost soundless within the frequencies of our interest. For a more intuitive presentation, we extract the data on the vertical dashed line at 7.46 kHz in Fig. 3c and replot the pressure amplitude distribution on a ring in Fig. 3d. The measured field distribution in Fig. 3d agrees well with the simulated result in Fig. 2b, thus validating the nearly unitary coupling efficiency. In Fig. 3d, the pressure amplitude field in the lower half ring manifests inevitable attenuation. Such attenuation mainly stems from thermo-viscous loss in the metamaterial waveguide with subwavelength slit arrays. The ratio between the pressure amplitudes at 90° and 270° of the coupling ring is illustrated in Fig. 3e, which is plotted by using the data on horizontal dashed lines in Fig. 3c. It clearly shows that the measured pressure amplitude contrast is above 20 from 7.44 to 7.48 kHz and reaches the maximum value of around 42 at 7.46 kHz. We find out that the measured contrast agrees well with the simulated one, as shown in Fig. 3e. After studying the properties of U-Ring-U shape waveguide complex, we can further design a 2D AFI and investigate the topological edge states in the strong coupling regime.

### Topological wave transportation in acoustic AFI

Basically, in the strong coupling regime, the direction of propagation and pseudo-spin of acoustic waves are locked at the boundaries of AFI. For example, the upper edge supports forward propagating waves with pseudo-spin-up and backward propagating waves with pseudo-spin-down, and conversely for the lower edge. In this section, we will numerically and experimentally display varied topological wave transportations, such as one-way edge states and interfacial states, in the acoustic AFI of different lattice configurations. The simulations are operated in lossless systems. It is necessary to emphasize that those phenomena are observable even in the presence of decay processes as revealed in experiments.

**Figure 4:**
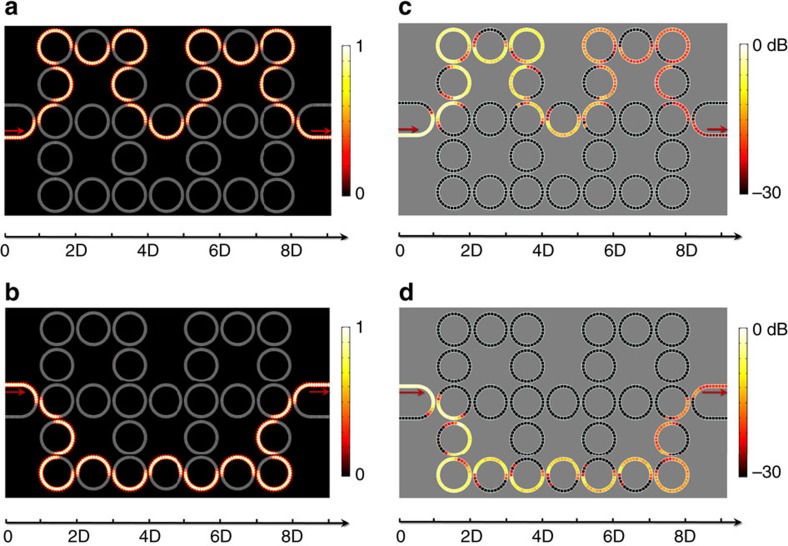
The simulated and measured pressure amplitude distributions of one-way edge states. (**a**,**b**) The simulated pressure amplitude distributions when pseudo-spin-up and pseudo-spin-down acoustic one-way edge states are selectively excited. We can clearly observe the robustness of the edge states against the sharp bending of the boundaries. (**c**,**d**) The measured sound pressure distributions, corresponding to **a** and **b**, respectively. The operating frequency of sound is 7.46 kHz. The distance between neighbouring lattice rings is 2*D*.

The first demonstration is pseudo-spin-dependent topological transportation of edge states in a lattice composed of 3 × 4 unit cells. One ring is purposely removed to form a dented edge as shown in Fig. 4. U-shape waveguides are imposed to selectively excite or extract pseudo-spin-up or pseudo-spin-down acoustic edge states. In Fig. 4a, we launch guided waves from the lower arm of the left U-shape waveguide and the pseudo-spin-up edge state is perfectly excited. The excited edge state is circulating clockwise in each lattice ring and immune to the dented edge. Therefore, it propagates through six sharp corners of the lattice smoothly. Owing to the nearly unitary coupling at each connection, the backward reflection of pseudo-spin-down component is negligible, leading to the fact that the output sound energy is closely equal to the input. However, when we launch guided waves from the upper arm, we will instead excite the pseudo-spin-down edge state, as shown in Fig. 4b. The guided wave in each lattice ring is circulating anti-clockwise and propagates along a different route with only two sharp corners. It should be mentioned that the pseudo-spin-dependent topological transportation of edge states even holds after weak lattice distortion is imposed (see Supplementary Fig. 4). We conduct the proof-of-concept experiment in an anechoic chamber. To prevent unwanted radiation losses during the propagation, the fabricated metamaterial ring lattice is sealed in rigid rectangular pipes and pressure amplitude distribution in the ring lattice is measured by inserting a condenser microphone into perforated holes on the pipe wall (see Supplementary Fig. 5). During the measurement, the lab-made sound source is operating at 7.46 kHz and the unscanned holes are bolted to prevent sound leakage (see Supplementary Figs 6 and 7). The measured pressure amplitude distributions for pseudo-spin-up and pseudo-spin-down acoustic one-way edge states are respectively shown in Fig. 4c,d, which agrees well with the simulated ones despite of obvious field attenuation. The significant dissipation is mainly due to thermo-viscous damping effect in the narrow air channels between metal plates. The details of experimental measurements are referring to the Supplementary Note 3. Owing to the perfect localization of edge states, the number of unit cells in the bulk region has no influence on the property of edge states. In the Supplementary Fig. 8, we have calculated band structures for the lattices with numbers of unit cells *N*=3, 5, 10, 20, 50 and 100 in the *y* direction. The result clearly shows that the edge state bands, *viz*., the red and blue curves, are unchanged as the number of unit cells *N* in *y* direction increases. The number *N* only influence the bulk state bands very much. For example, as *N* increases, the number of bulk state bands also increases. In this work, we choose the lattice of 3 × 4 due to the limitation of experimental conditions. However, our results still unequivocally show the existence of topological edge states in the confined system.

**Figure 5:**
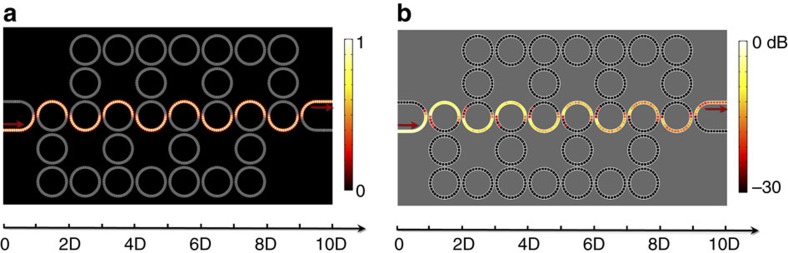
The simulated and measured pressure amplitude distributions of the one-way interface state. (**a**) The simulated pressure amplitude distribution for the interface state after the lattice is dislocated by half a lattice constant *D*. The pseudo-spin-up interface state is excited from the lower arm of the left U-shape waveguide and propagates unidirectionally to the right output. If we excite the wrong spin component at the input port, we will obtain a bulk state instead of an interface state (see Supplementary Fig. 9). (**b**) The measured sound energy distribution for this one-way interface state. The operating frequency of sound is 7.46 kHz.

In the next, we demonstrate the existence of non-trivial interface states in a dislocated lattice, where two sub-lattices (2 × 4 unit cells) are horizontally dislocated by half a lattice constant. Simulation in Fig. 5a unequivocally shows that the interface state launched at the left can propagate along the dislocated interface to the right output with little reflections. As the coupling and lattice rings are chosen to be of the same size, the lower and upper sub-lattices can match well with each other at the interface under the dislocation of half a lattice constant. Basically, the coupling ring of the lower sub-lattice acts as the lattice ring of the upper sub-lattice and vice versa. Thus, the interface state can be regarded as pseudo-spin-down edge state for the upper sub-lattice and pseudo-spin-up edge state for the lower one, respectively. Differing from the edge states that can be excited from either the lower branch or the upper branch of the U-shape waveguide at the left side, the interface state can only be excited from the lower branch of the U-shape waveguide. If we launch acoustic waves from the upper branch, we will excite the bulk state instead of interface state (see Supplementary Fig. 9). It shows that the field distributions of interface state and bulk state are complementary to each other in the dislocated lattice. In Fig. 5b, we provide the measured sound energy distribution of the interface state, where the interface state can propagate from the left to right with little reflection. Owing to the presence of loss, the sound is attenuated exponentially in the propagation.

**Figure 6:**
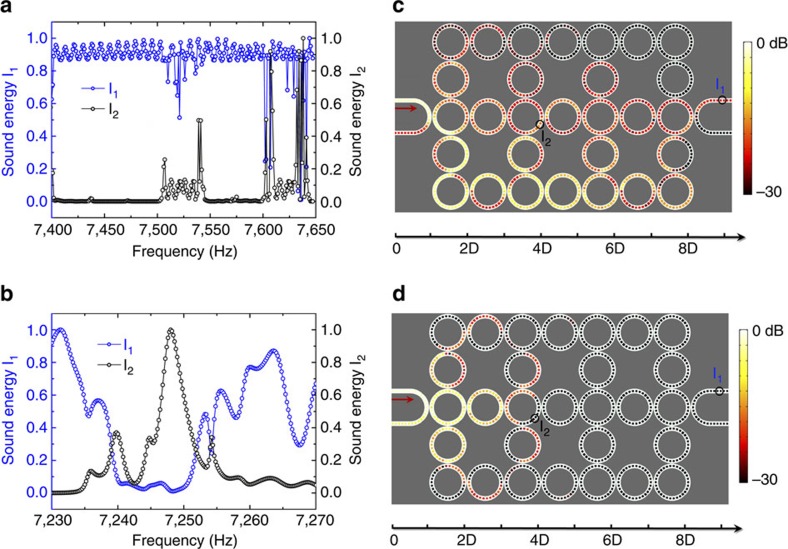
The measured sound energy spectra and sound energy distributions of pass band and band gap states. (**a**,**b**) The measured sound energy spectra in the frequency ranges of 7.4∼7.65 kHz and 7.23∼7.27 kHz, respectively. In the experiments, we measured the sound energy spectra (*I*_1_ and *I*_2_) at two different sites, as marked by the circles in **c**,**d** (also see Supplementary Fig. 10). *I*_1_ and *I*_2_ are normalized with respect to their maxima (arb. unit). In light of the amplitudes of *I*_1_ and *I*_2_, we can clearly observe the bandwidths of gapless edge states, pass bands, as well as band gaps. (**c**,**d**) The measured sound energy distributions in the pass band (7.526 kHz) and the band gap (7.247 kHz), respectively.

At last, the properties of pass band and band gap states are investigated by measuring the sound energy spectra and sound energy distributions in AFI. In Fig. 6a,b, the sound energy spectra (*I*_1_ and *I*_2_) are shown to check the bandwidths of the edge states. In the experiments, *I*_1_ and *I*_2_ are measured at two different sites, as marked by the circles in the Supplementary Fig. 10. *I*_1_ indicates the sound energy at the output, whereas *I*_2_ reflects the sound energy penetrated into the bulk. *I*_1_ and *I*_2_ are normalized with respect to their maxima (arbitrary unit). In principal, we can determine the edge states, pass band states and band gap states from the amplitudes of *I*_1_ and *I*_2_. Specifically, *I*_1_>0, *I*_2_→0 for the edge states, *I*_1_>0, *I*_2_>0 for the pass band states and *I*_1_→0 for the band gap states. From the provided criterion, one can easily obtain that the carrying frequencies of edge states span from 7.4 to 7.5 kHz and from 7.55 to 7.6 kHz, as shown in Fig. 6a, which agrees well with the band structure diagram in Fig. 2c. We also obtain that the bulk band and the band gap span from 7.5 to 7.55 kHz and from 7.24 to 7.25 kHz, respectively. From the above, the bandwidths of gapless edge states below and above the bulk band are thus calculated to be around 100 and 50 Hz, respectively. Figure 6c,d provide the measured sound energy distributions in the pass band (7.526 kHz) and the band gap (7.247 kHz). In the pass band, the sound energy is distributed not only at the edge but also in the inside bulk. In the band gap, the sound energy is rapidly decayed in the *x* direction. Thus, the sound is completely blocked by the lattice array. We find out that the experimental results fairly agree with the numerical simulations, after comparing Fig. 6 with Supplementary Fig. 11. It is also worthy to be pointed out that the gapless edge states below and above the bulk bands share similar topological properties. Although their bandwidths are quite different, the sound energy distributions are nearly the same, as shown by the experimental results in Supplementary Fig. 12.

## Discussion

In summary, we have experimentally demonstrated the prototype of *T*-invariant AFI for sound, which permits unidirectionally propagating pseudo-spin-dependent edge states. Here we would like to mention the works by Hafezi *et al*.^7^,^8^, where the coupling rings are aperiodic and well designed for purposely constructing an effective magnetic field to mimick the quantum Hall effects. In stark contrast with the work by Hafezi *et al*.^7^,^8^, our proposed AFI is not only periodic but also featured with the same size of lattice rings and coupling rings. This particular size selection is beneficial for the experimental demonstration, as the processing difficulty of the sample is greatly reduced and the required distribution of the coupling strength between neighbouring rings can be well satisfied in practice. In addition, we have discovered a novel topological interface state after introducing the dislocation of half a lattice constant into AFI. It should be emphasized that all the coupled rings need to be identical for the best excitation of such dislocation-induced topological interface state. It is also necessary to be mentioned that the *T*-symmetry between electrons and acoustic waves have fundamentally different physical natures. For example, eigenvalues of *T*^2^ operator for electrons *T*^2^=−1 and for acoustic waves *T*^2^=1 (ref. 24). As a result, electrons with different spins are completely decoupled under the protection of *T*-symmetry, whereas acoustic waves with different pseudo-spins can easily scatter into each other through time-reversed channels, such as imperfect couplings. To solve the backscattering issue in acoustic AFI, it is meaningful to push the coupling up to unitary and make acoustic waves with different pseudo-spins nearly decoupled. Another possible way of eliminating backscattering is breaking *T*, for example, using well-designed time-varying modulations^18^,^27^,^28^, which however is experimentally challenging and beyond the scope of this work.

Our work provides a fertile ground for novel wave manipulations, such as the unidirectional sound transports and robust sound transports against perturbations, and pushes forward the fundamental explorations of topological acoustics. Of interest will be the extension of our work into non-reciprocal acoustics regime by integrating time-varying (for example, rotating the metamaterial rings), shedding lights on the development of chiral acoustic metamaterials and Chern acoustic topological insulators with various intriguing non-reciprocal properties, such as one-way sound isolation.

## Methods

### Sample preparation

We fabricated the waveguide metamaterial with aluminum alloys plates. Periodic grooves are perforated on the plate by numerically controlled machine tools (accuracy: 0.1 mm) with period 4.3 mm, width 17.2 mm and thickness 2.6 mm, whereas the height of each groove is constant (10 mm) and much smaller than the wavelength of sound in air (∼46 mm at 7.46 kHz). For the coupled metamaterial ring lattice, the inner and outer radii are 77.4 and 94.6 mm for each ring, respectively, distance of centres of nearest-neighbour rings is 198 mm and the angle between adjacent grooves in each ring is 3°.

### Experiments setup and measurements

In the experiment, a lab-made sound source driven by a multifunctional signal generator (SRS MODEL DS345) and a lab-made power amplifier was placed in front of the tapered end of the metamaterial waveguide, to generate stable waveguide modes (see Supplementary Fig. 6). The sound energy is measured through a 1/8-inch diameter Brüel&Kjær Type-LAN-XI-3160 condenser microphone. The data are recorded with Brüel&Kjær PULSE 3160-A-042 4-channel analyser. The frequency response is obtained with fast fourier transform (FFT) analysis of Brüel&Kjær PULSE software LabShop version 19.0.0.128. To efficiently suppress the unwanted back reflections at input (or output) facets, we use gradient metamaterials with acoustic impedance fairly matched to air in broadband (see Supplementary Fig. 13). The fabricated metamaterial waveguides are all sealed in rigid rectangular pipes, to prevent unwanted radiation losses during propagation. Pressure amplitude distribution in the ring lattice is measured by inserting a condenser microphone into perforated holes on the pipe wall, where the perforated holes are azimuthally periodic and segregated by 12°. In each measurement, the unscanned holes are bolted to prevent sound leakage.

### Numerical calculations

We employ COMSOL MultiphysicsTM 4.3b to perform FEM simulations. The geometrical model of the metamaterial waveguide complex is built up in the commercial software Pro/Engineer and then is loaded into the acoustic–solid interaction multi-physics module for the full-wave simulations. The materials applied in simulations were air and Aluminum (6061 T6). Perfectly matched layers are imposed on the outer boundaries of simulation domains to prevent reflections. The largest mesh element size was set lower than 1/10 of the lowest wavelength and finer meshes were applied at the domain with abrupt geometry changes.

### Data availability

The data that support the findings of this study are available from the corresponding author upon request.

## Additional information

**How to cite this article**: Peng, Y.-G. *et al*. Experimental demonstration of anomalous Floquet topological insulator for sound. *Nat. Commun.* **7**, 13368 doi: 10.1038/ncomms13368 (2016).

**Publisher's note:** Springer Nature remains neutral with regard to jurisdictional claims in published maps and institutional affiliations.

## Supplementary Material

Supplementary Information Supplementary Figures 1-14, Supplementary Notes 1-3 and Supplementary References
