# Supplementary material for: Experimental demonstration of anomalous Floquet topological insulator for sound
Source: Nat Commun. 2016 Nov 11;7:13368. doi: 10.1038/ncomms13368 (PMC5114601; doi:10.1038/ncomms13368)
Supplement: Supplementary Information — Supplementary Figures 1-14, Supplementary Notes 1-3 and Supplementary References [file ncomms13368-s1.pdf]

SUPPLEMENTARY FIGURES

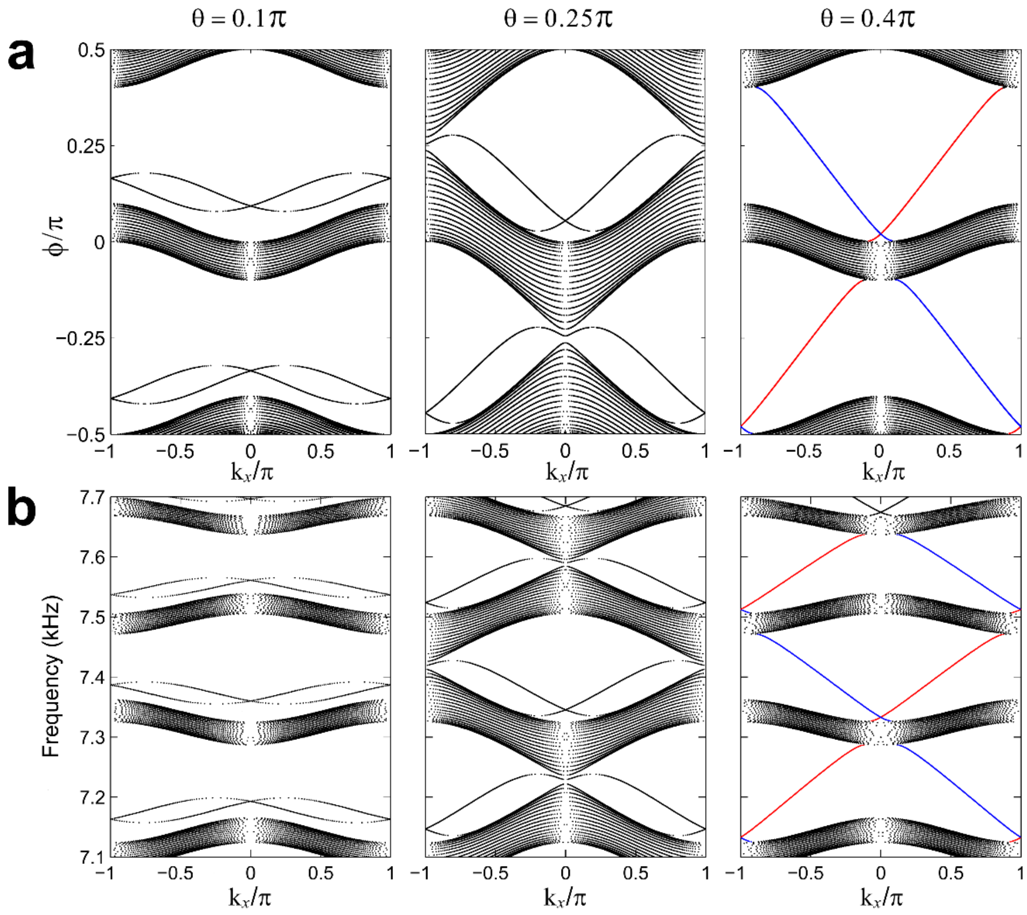

Supplementary Figure 1. | Projected band structures for different coupling strengths. (**a**) The non-dispersive quasi-energy diagrams and (**b**) projected band structures for constant coupling strengths of θ=0.1π, 0.25π, and 0.4π, respectively. The number of unit cells in the transverse (*y*) direction is fixed as *N*=20. Here, the condition of θ=0.1π (<0.25π) corresponds to the weak coupling regime, where the ring lattice is a trivial insulator (no gapless edge states). The condition of θ=0.4π (>0.25π) corresponds to the strong coupling regime, where that the ring lattice is a topological insulator (a pair of gapless edge states at upper and lower boundaries of the lattice, marked by the red and blue curves). θ=0.25π denotes the transition point between the weak and strong coupling regimes. The numerical calculations of different non-dispersive cases are in excellent agreements with the results in Ref. 11 and 30.

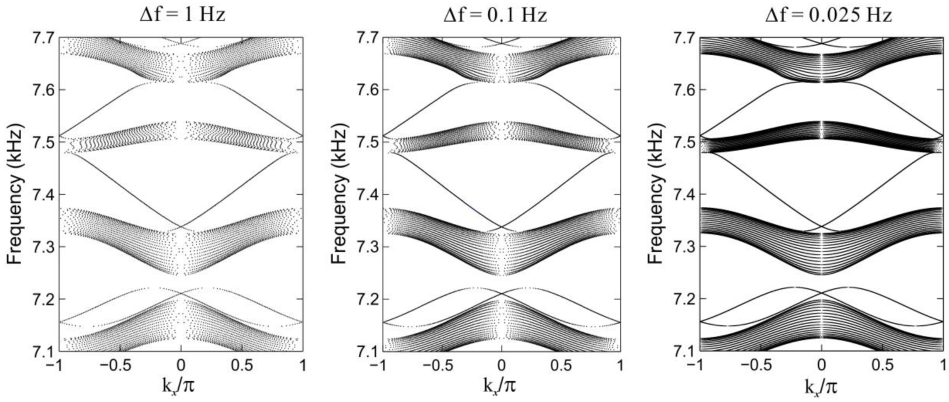

Supplementary Figure 2. | Projected band structures for different frequency steps. The projected band structures for different frequency steps, *viz*., Δ*f*=1Hz, 0.1Hz, and 0.025Hz, where the frequency is scanned from 7.1kHz to 7.7kHz. The number of unit cells in the transverse (*y*) direction is fixed at *N*=20.

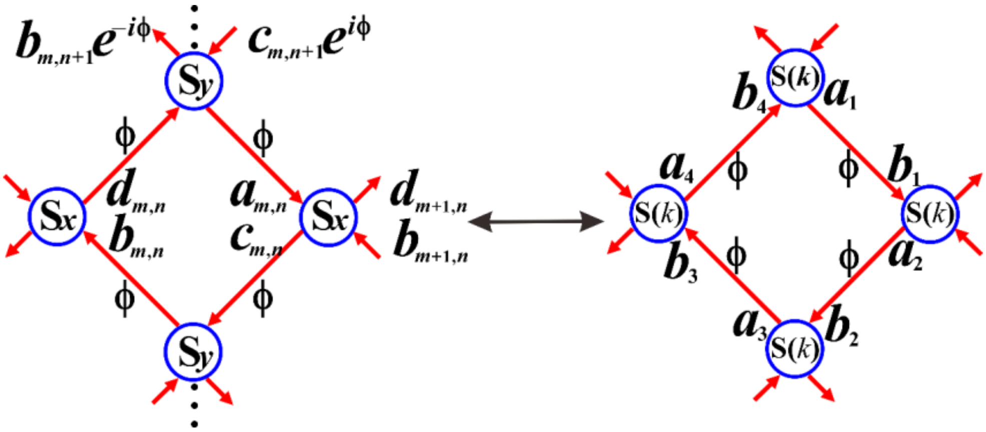

Supplementary Figure 3. | The Chalker-Coddington network model. The left hand side shows one unit cell of the Chalker-Coddington network model of the coupled ring lattice, while the right hand side is the scattering matrix model of one unit cell for a fixed Bloch vector *k*.

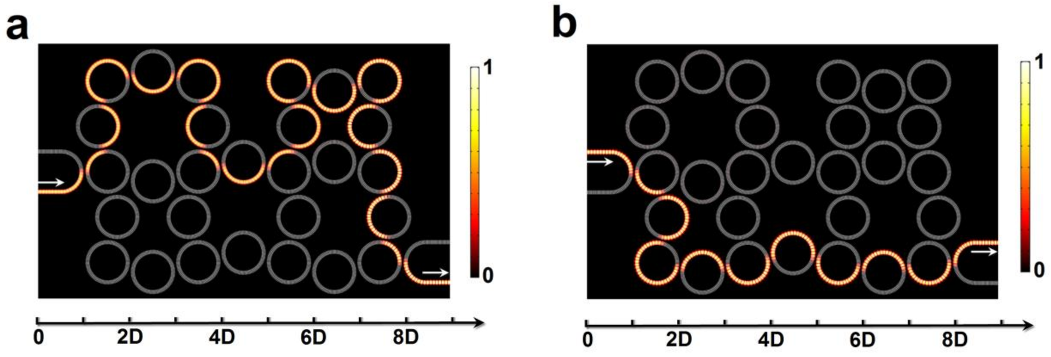

Supplementary Figure 4. | Acoustic topological insulator with a distorted lattice. (**a**) and (**b**) The simulated acoustic amplitude distributions when pseudo-spin-up and pseudo-spin-down acoustic one-way edge states are selectively excited. Here, the 2D lattice is arbitrarily distorted but couplings between the adjacent rings remain unitary. Our simulation clearly shows that the pseudo-spin-dependent acoustic one-way edge state behaves more like a conventional waveguide mode, where the scatterings from boundary abrupt variations or lattice dislocations are barely observed.

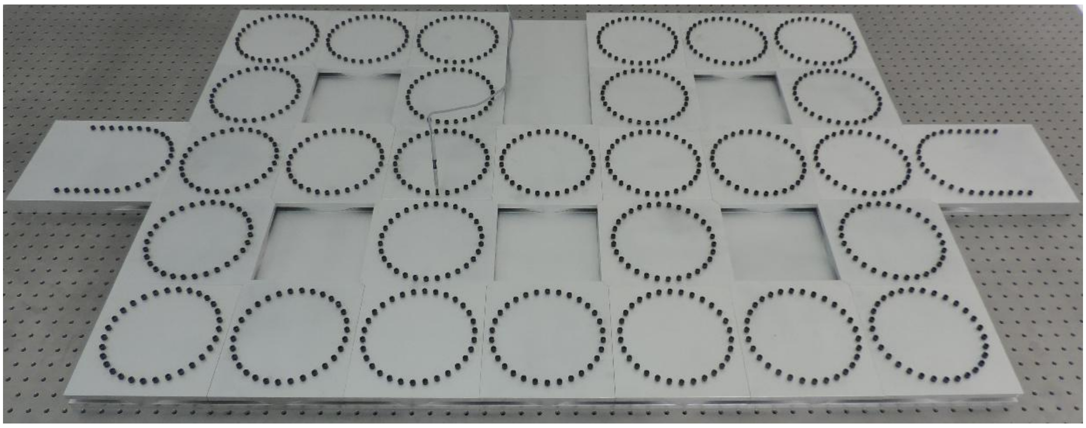

Supplementary Figure 5. | Illustration of the experimental measurement setup. The acoustic Floquet topological insulator, *viz*. 2D coupled metamaterial ring lattice, is sealed in rigid rectangular pipes to eliminate radiation losses. A condenser microphone is inserted into perforated holes on the pipe wall to measure the pressure amplitude in the metamaterial rings.

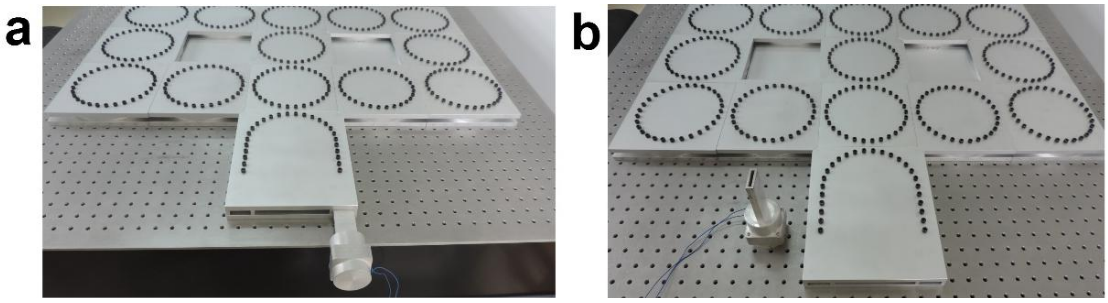

Supplementary Figure 6. | Zoom-in photograph of a lab-made sound source. In the experiment, the lab-made sound source driven by a multifunctional signal generator (SRS MODEL DS345) and a lab-made power amplifier was placed in front of the tapered end of the metamaterial waveguide to generate stable waveguide modes.

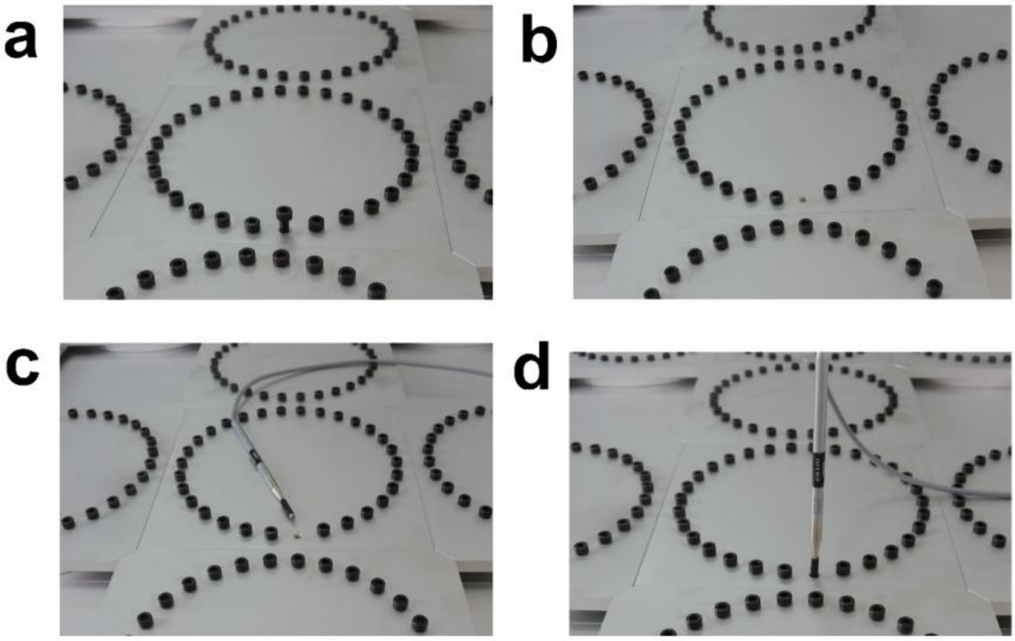

Supplementary Figure 7. | Zoom-in photograph of the metamaterial ring. During the measurement, the condenser microphone is inserted into a hole to extract the pressure amplitude inside, while the unscanned holes are bolted to prevent sound leakage.

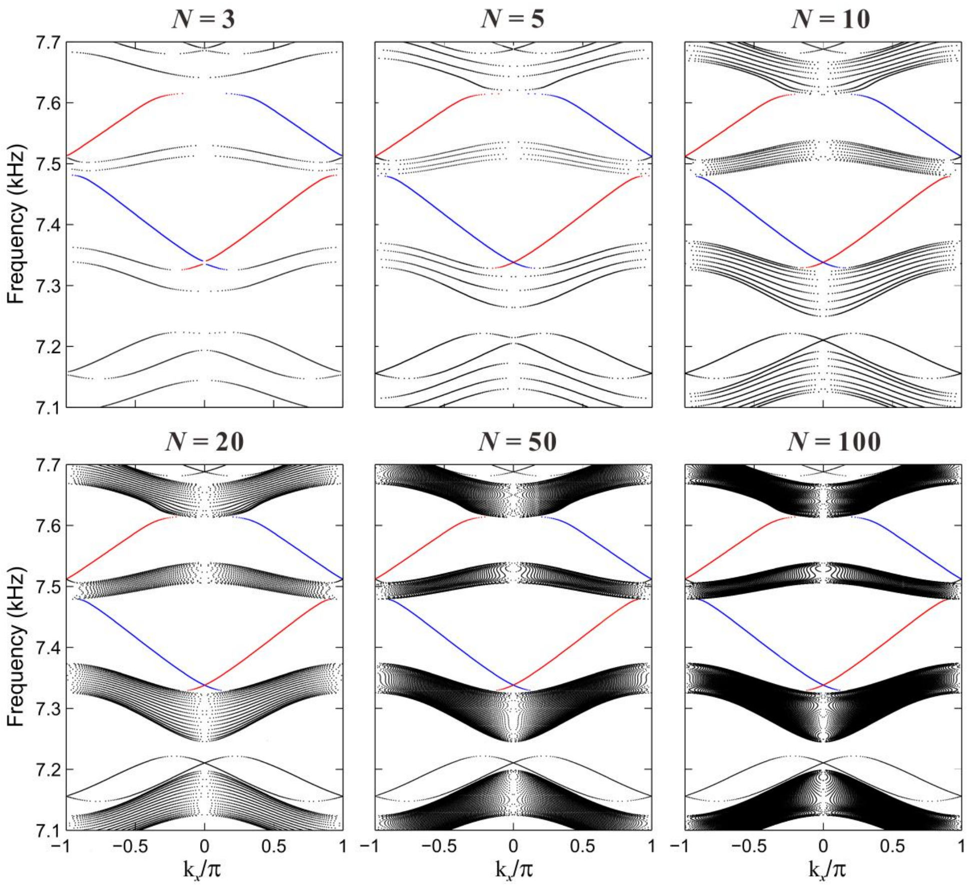

Supplementary Figure 8. | Projected band diagrams for different lattices. Projected band diagrams for different semi-infinite strip lattices with unit cells of *N*=3, 5, 10, 20, 50, and 100 in the transverse (*y*) direction.

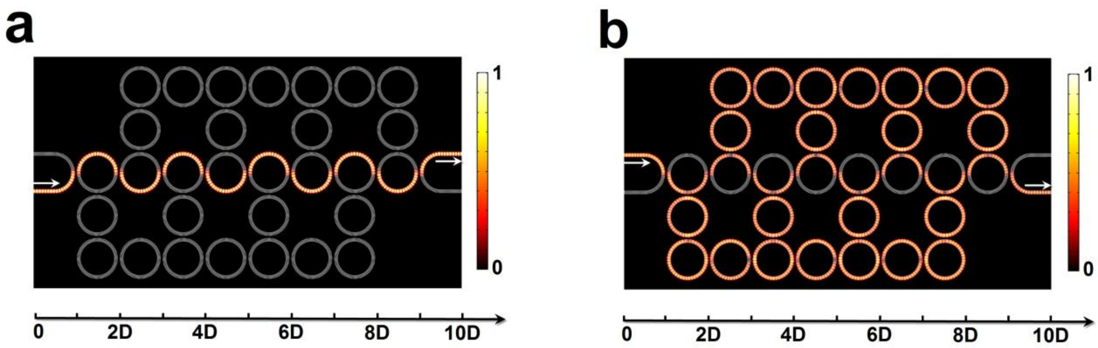

Supplementary Figure 9. | Acoustic one-way interface state. After introducing a lattice dislocation by half a lattice constant, we obtain a novel acoustic one-way interface state in (**a**), propagating from left to right for the pseudo-spin-up component, and conversely for the pseudo-spin-down component. If we excite the wrong pseudo-spin component at the left input port, we will obtain a bulk state with the pressure amplitude distribution interestingly being complementary to that of the interface state, as shown in (**b**).

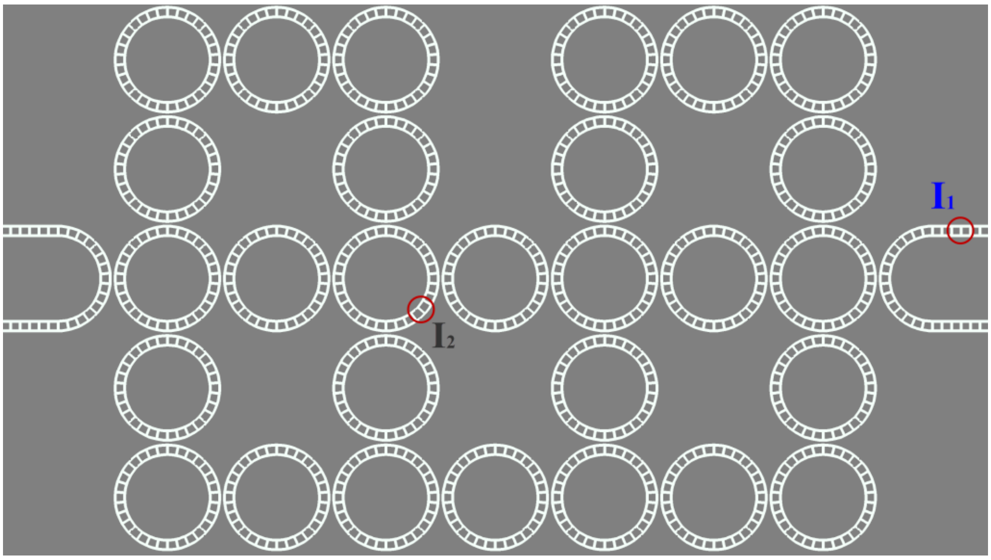

Supplementary Figure 10. | Positions for energy spectra measurement. The circles show the positions where we measured the sound energy spectra (I_1 and I_2). Each circle covers two sub-sections that correspond to two holes perforated on the waveguide ring. The sound energy spectra I_1 and I_2 are obtained from the average of the sound energy data extracted from the two holes.

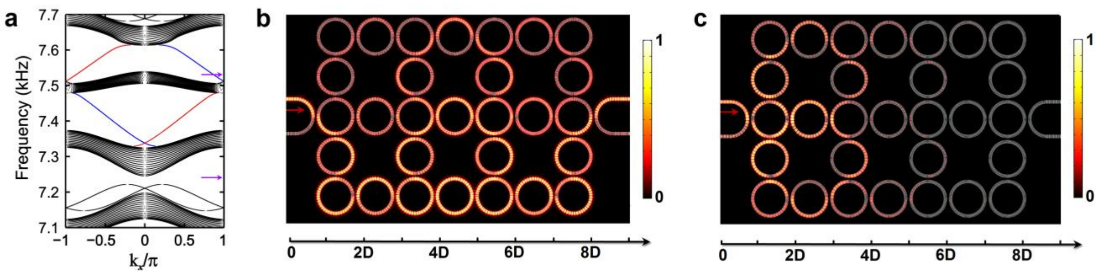

Supplementary Figure 11. | Simulated results for pass band and band gap states. (**a**) Projected band structure of the semi-infinite ring lattice (*N*=20) for pseudo-spin-up Bloch modes. The red and blue bands denote nontrivial edge states at upper and lower boundaries of the lattice. The purple arrows mark the frequencies of 7.526kHz and 7.247kHz. (**b**) and (**c**) The simulated pressure amplitude distributions in the pass band (7.526kHz) and the band gap (7.247kHz), respectively.

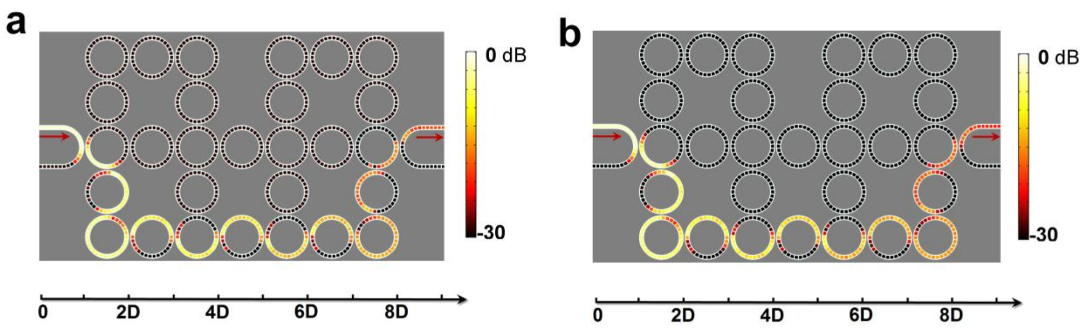

Supplementary Figure 12. | Experimental results for edge states. (**a**) and (**b**) The measured sound energy distributions of the edge states located at 7.46kHz and 7.58kHz, respectively.

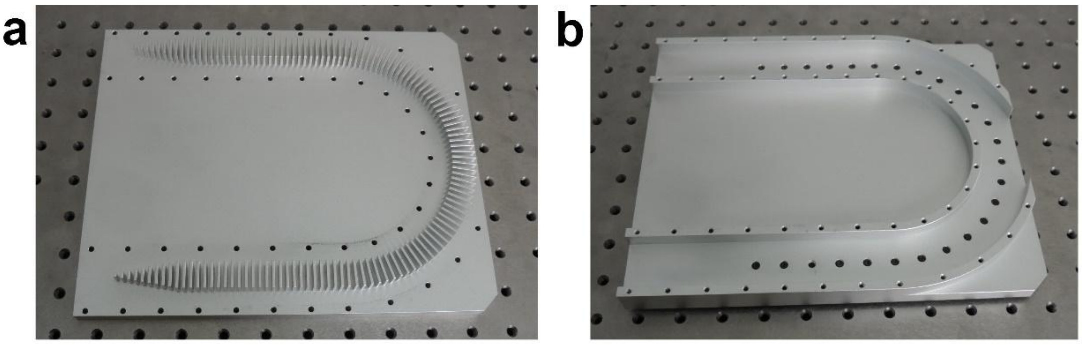

Supplementary Figure 13. | Illustration of gradient metamaterials. To efficiently suppress the unwanted back-reflections at input (or output) facets, we use gradient metamaterials with acoustic impedance fairly matched to air in broadband.

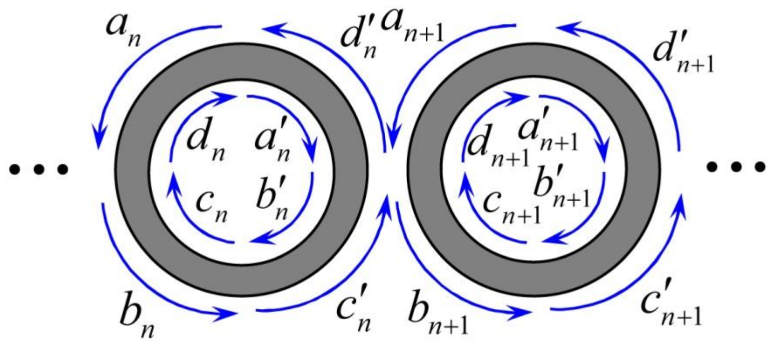

Supplementary Figure 14. | A periodic chain of coupled ring resonators. In the schematic diagram, the clockwise and anti-clockwise propagating field components are labeled for theoretical analysis in Supplementary Note 1.

SUPPLEMENTARY NOTES

Supplementary Note 1. The model of a periodic 1D chain of coupled ring resonators. To analyze the model of a periodic 1D chain of coupled ring resonators, we at first define a vector with the field components labeled in Supplementary Figure 14, *viz.* p_n=[a_n b_n c_n d_n]'. The scattering process at each coupling connection can be described by

$$\begin{bmatrix} b'_n \\ b_{n+1} \end{bmatrix} = \begin{bmatrix} t & \kappa \\ -\kappa^* & t^* \end{bmatrix} \begin{bmatrix} a'_n \\ a_{n+1} \end{bmatrix}, \quad \begin{bmatrix} d'_n \\ d_{n+1} \end{bmatrix} = \begin{bmatrix} t & \kappa \\ -\kappa^* & t^* \end{bmatrix} \begin{bmatrix} c'_n \\ c_{n+1} \end{bmatrix}, \tag{1}$$

From Supplementary Equation (1), we can relate p_n with p_{n+1} through[1]

$$p_{n+1} = \begin{bmatrix} M_1 & 0 \\ 0 & M_1 \end{bmatrix} \begin{bmatrix} 0 & M_2 \\ M_2 & 0 \end{bmatrix} p_n, \tag{2}$$

where

$$M_1 = \frac{1}{\kappa} \begin{bmatrix} -t & 1 \\ -1 & t^* \end{bmatrix} \text{ and } M_2 = \begin{bmatrix} 0 & e^{-i\beta R\pi} \\ e^{i\beta R\pi} & 0 \end{bmatrix}.$$

In Supplementary Equation (2), R is the ring radius and $\beta=n_{eff}\omega/c_{air}$ is the wave number without considering the loss. For the periodic chain of coupled ring resonators (lattice constant Λ), we can apply the Bloch's theorem[2]

$$p_{n+1} = \exp(-iK\Lambda)\, p_n, \tag{3}$$

where K is the Block mode wave number. Comparing Supplementary Equation (3) with Supplementary Equation (1), we obtain that Bloch modes of the periodic 1D chain of coupled ring resonators can be described by the eigenvectors of

$$M = \begin{bmatrix} M_1 & 0 \\ 0 & M_1 \end{bmatrix} \begin{bmatrix} 0 & M_2 \\ M_2 & 0 \end{bmatrix}. \tag{4}$$

At the frequency ω, the eigenvalue E_n and eigenvector V_n are

$$E_1 = \exp(-iK_1\Lambda) \text{ and } V_1 = [\lambda_1 + \lambda_2 \quad 1 \quad \lambda_1 + \lambda_2 \quad 1]',$$

$$E_2 = -\exp(-iK_1\Lambda) \text{ and } V_2 = [-\lambda_1 - \lambda_2 \quad -1 \quad \lambda_1 + \lambda_2 \quad 1]',$$

$$E_3 = \exp(-iK_2\Lambda) \text{ and } V_3 = [\lambda_1 - \lambda_2 \quad 1 \quad \lambda_1 - \lambda_2 \quad 1]',$$

$$E_4 = -\exp(-iK_2\Lambda) \text{ and } V_4 = [-\lambda_1 + \lambda_2 \quad -1 \quad \lambda_1 - \lambda_2 \quad 1]', \tag{5}$$

with

$$\lambda_1 = \frac{1}{2t}\sqrt{1 + \exp\left(\frac{-i4m\pi\omega}{\Omega}\right) + 2\exp\left(\frac{-i2m\pi\omega}{\Omega}\right)\left(1 - 2t^2\right)},$$

$$\lambda_2 = \frac{1}{2t}\left(1 + \exp\left(\frac{i2m\pi\omega}{\Omega}\right)\right),$$

where Ω is the resonant frequency of each ring resonator and m is the order of whispering gallery mode ($m=\Omega n_{eff}R/c_{air}$). On condition of strong coupling $|\kappa|\rightarrow 1$, we will have $\omega\approx\Omega$, $t<<1$, $\lambda_1+\lambda_2\approx 2/t$, and $\lambda_1-\lambda_2\approx 0$. The eigenvectors in Supplementary Equation (5) are further simplified into

$$V_1 \approx [2/t \quad 1 \quad 2/t \quad 1]', V_2 \approx [-2/t \quad -1 \quad 2/t \quad 1]',$$

$$V_3 \approx [0 \quad 1 \quad 0 \quad 1]', V_4 \approx [0 \quad -1 \quad 0 \quad 1]'. \tag{6}$$

From Supplementary Equation (6), we find out that two field components are much more stronger than the other ones, which reveals that the wave will zigzag through the periodic chain of coupled ring resonators by making only half of a round trip in each ring. In this case, the rings no longer act like resonators but more close to the conventional waveguides.

Supplementary Note 2. None-zero v_1 invariant

For the periodic lattice in Supplementary Figure 3 (the left hand side), the Bloch modes propagate in the x direction, carrying a wave vector k. By assuming the coupling strength to be nondispersive, the projected band diagram turns into a Floquet one, denoted by $\phi(k)$ as shown in Refs. 11 and 30. Here, the factor ϕ is referred to as "quasi-energy", which represents the phase delay in each quarter ring. As shown in Supplementary Figure 3 (the right hand side), ϕ is the eigenvalue of the unitary scattering matrix $\mathbf{S}(k)$, satisfying the eigen-equation $\mathbf{S}(k)|b_k\rangle=e^{-i\phi}|b_k\rangle$, where the mode amplitudes in the nth unit cell and the corresponding Fourier components are expressed into $|b_n\rangle=(b_{n1}, b_{n2}, b_{n3}, b_{n4})^T$ and $|b_n\rangle=|b_k\rangle\exp(ikx_n)$, respectively. The factor ϕ is periodic, satisfying $\phi\equiv\phi+2\pi$. The periodic network with a Floquet band structure can be characterized by the v_1 invariant, which is defined by

$$v_1 = \frac{1}{2\pi}\int_{-\pi}^{\pi} dk \mathrm{Tr}[\mathrm{S}(k)^{-1} i\partial_k \mathrm{S}(k)], \tag{7}$$

Here, k is integrated over the first Brillouin zone. According to Refs. 11 and 30, the v_1 invariant is non-zero, which can be employed to describe the nontrivial topological properties of time-reversal invariant systems, *e.g.*, anomalous Floquet topological insulator.

Supplementary Note 3. Tips in the experimental measurements

To efficiently suppress the unwanted back-reflections at the input (or output) facets, we adopt the gradient metamaterials with the acoustic impedance fairly matched to air in broadband. According to the Equation (1) in the manuscript, the effective refractive index n_{eff} of the waveguide metamaterials is closely related to the waveguide width *w*. The gradient metamaterial structure is simply realized by adiabatically changing the waveguide width *w* as shown in Supplementary Figure 12.

In the experiment, the fabricated metamaterial waveguides were completely sealed in rigid rectangular pipes to prevent unwanted radiation losses during the propagation and isolate ambient noises (Supplementary Figure 5). To measure the pressure amplitude distribution at the metamaterial ring, we have drilled holes on the pipe walls. During the measurement, a 1/8 inch diameter Brüel&Kjær Type-LAN-XI-3160 condenser microphone was inserted into a hole to extract the pressure amplitude inside, while the unscanned holes were bolted to prevent sound leakage, as shown in the Supplementary Figure 7. To generate stable waveguide modes, we have designed and fabricated a rectangular sound source which was placed in front of the tapered end of U shape metamaterial waveguide, as shown in the Supplementary Figure 6.

SUPPLEMENTARY REFERENCES

1. Poon, J. K. S. *et al.* Matrix analysis of microring coupled-resonator optical waveguides. *Opt. Express* **12**, 90 (2004).

2. Yariv, A. & Yeh, P. *Optical waves in crystals: Propagation and control of laser radiation* (Wiley, New York, 1984).
